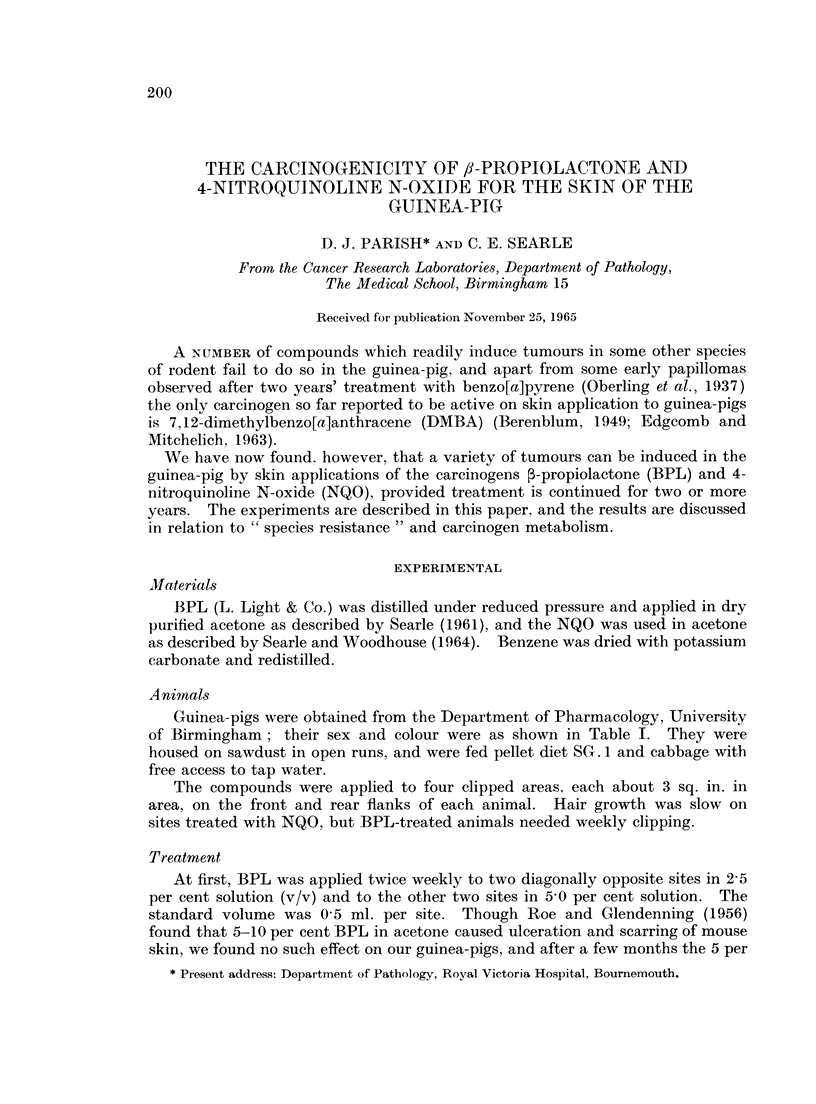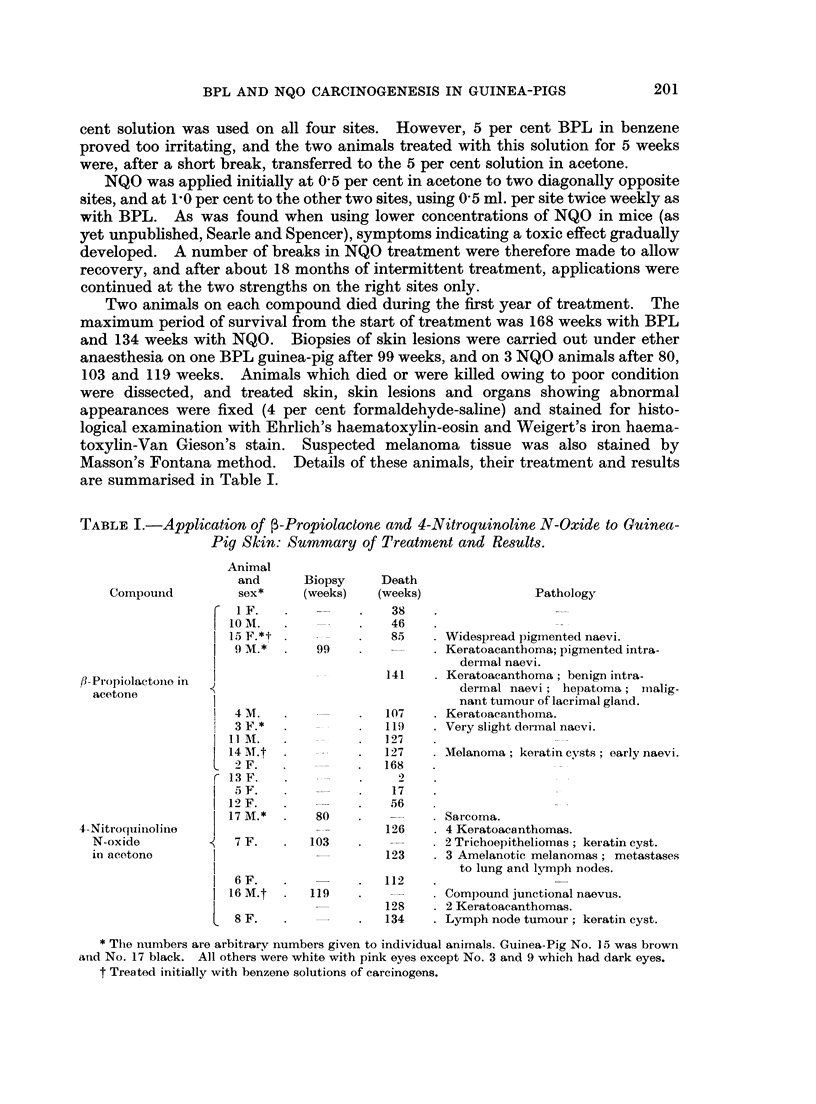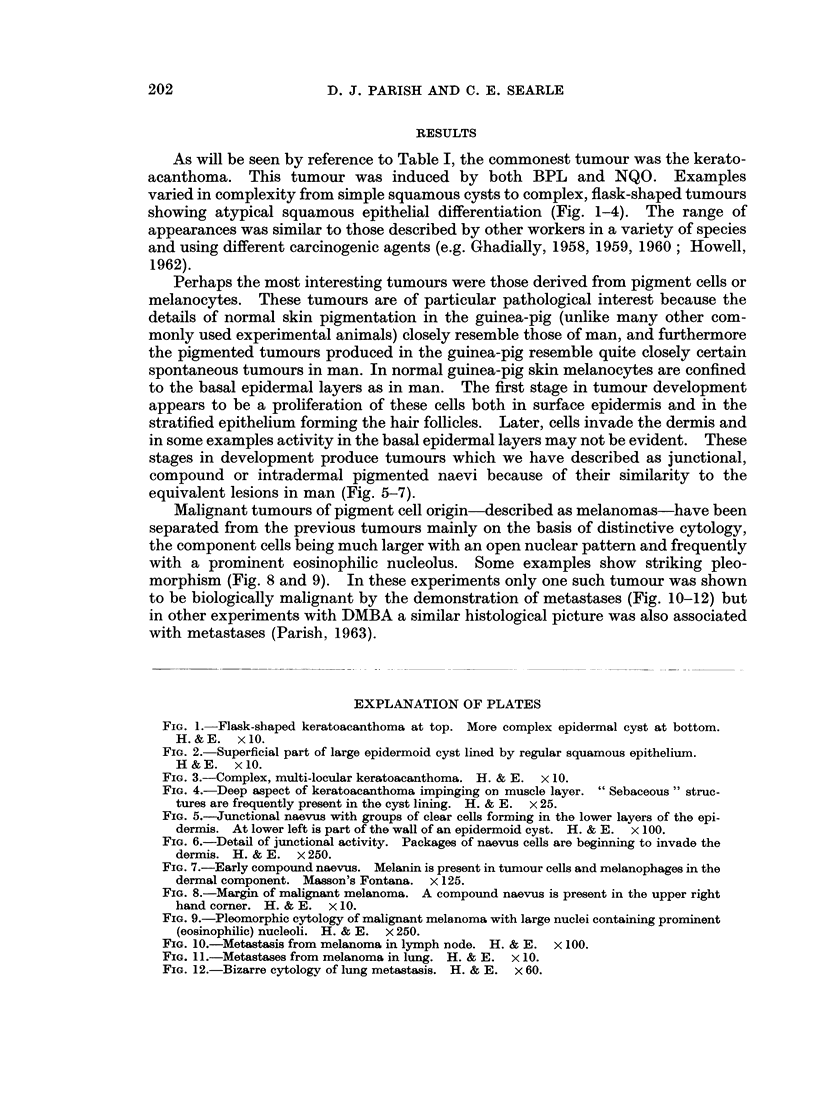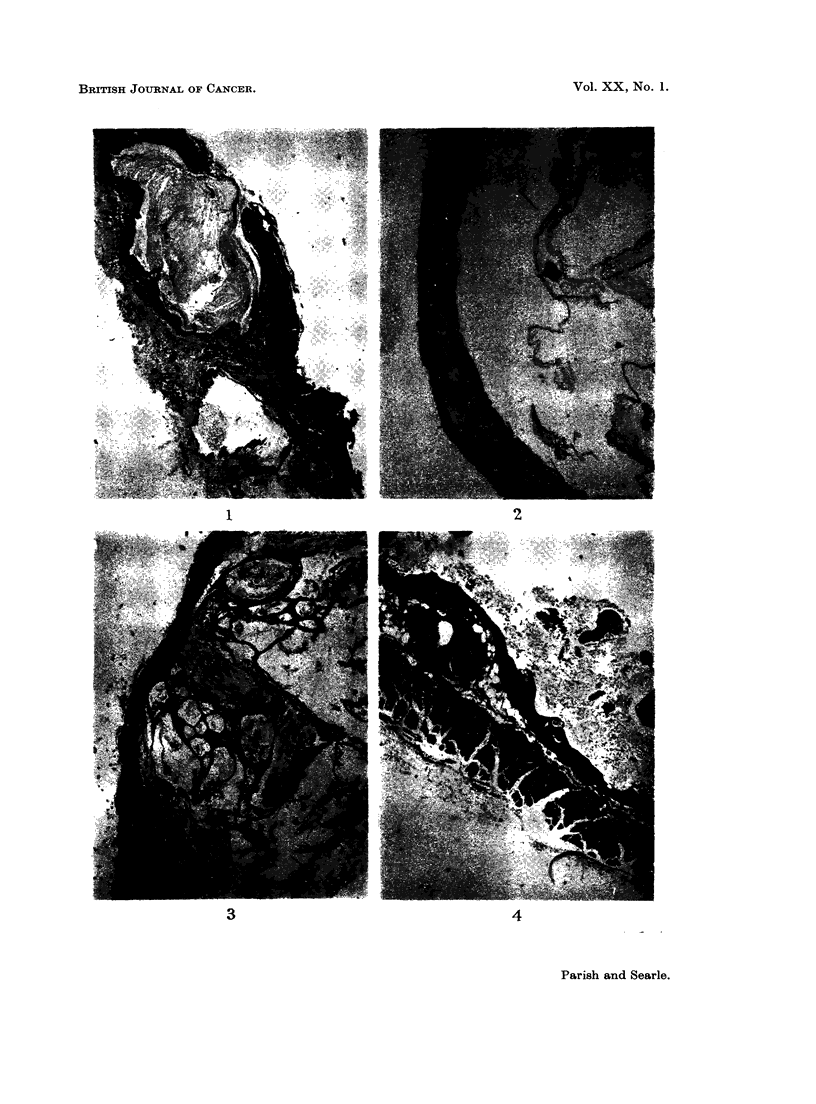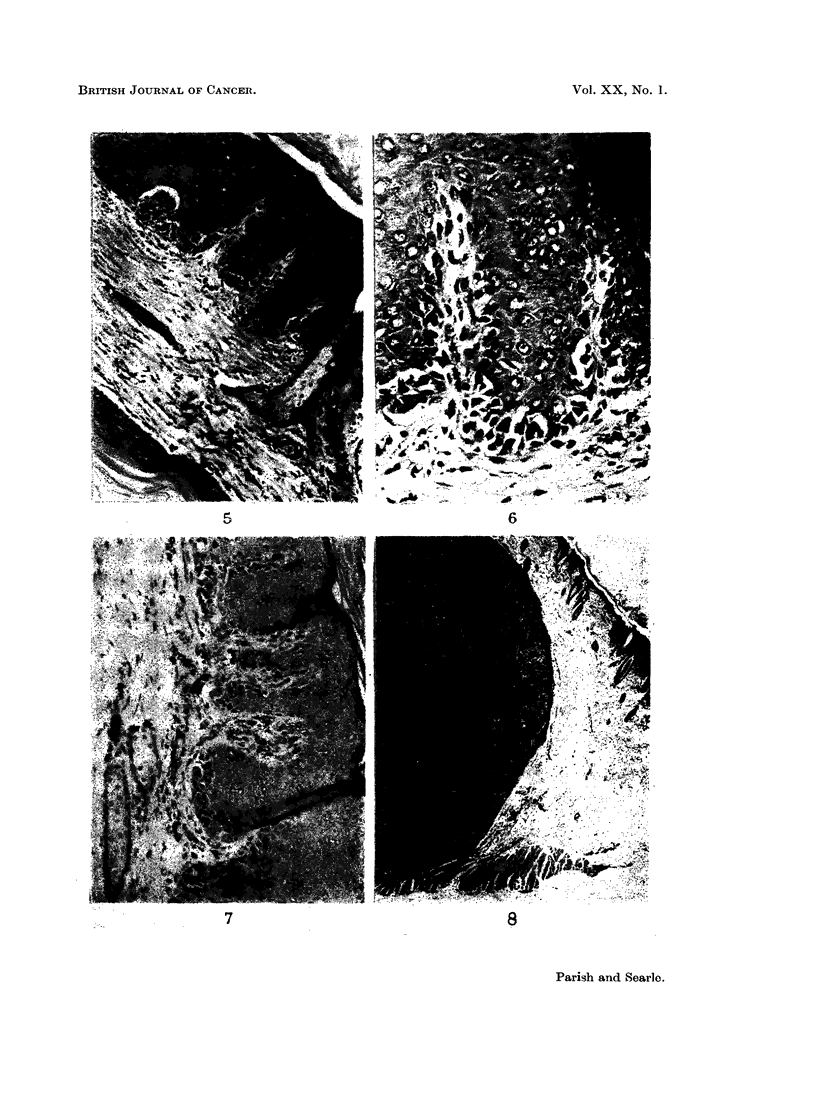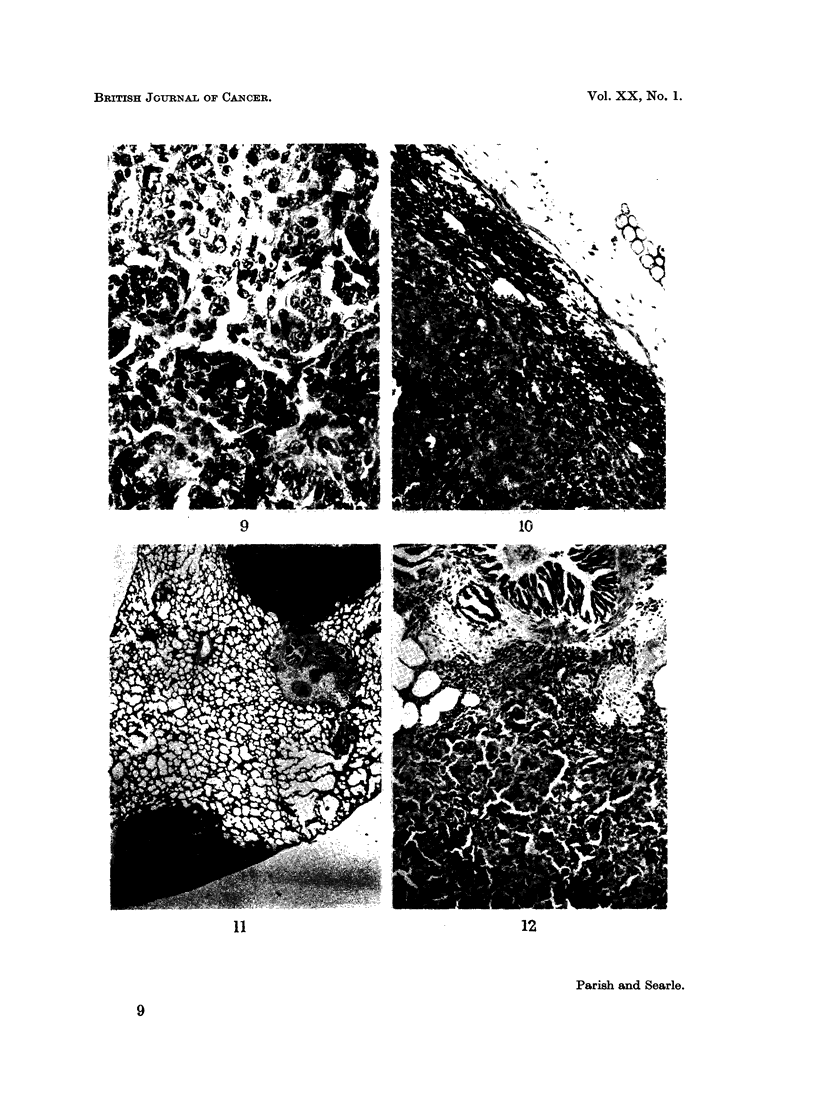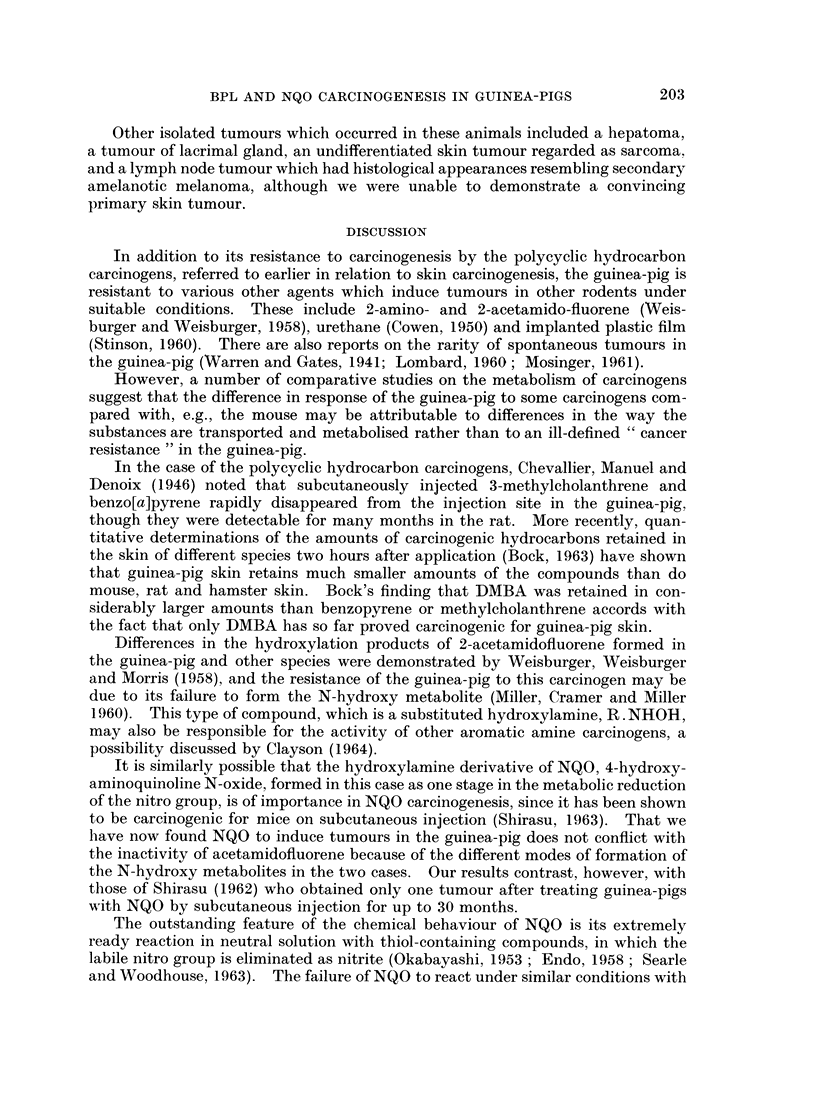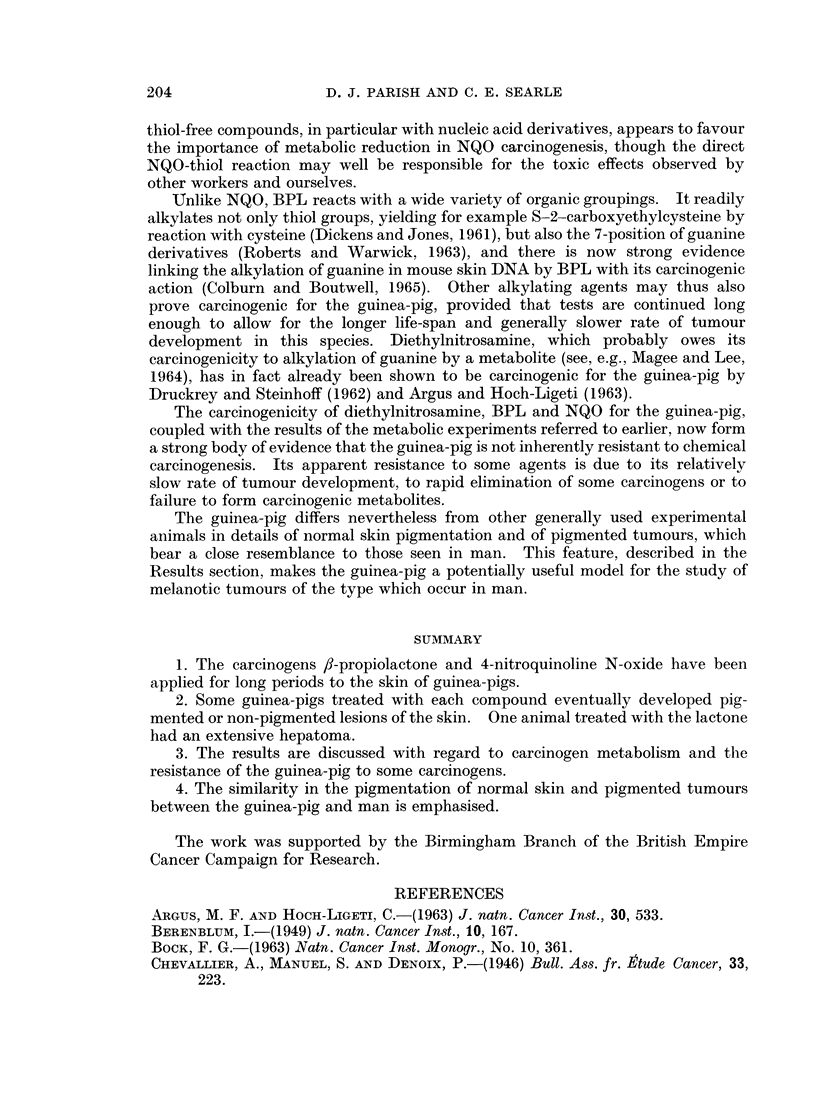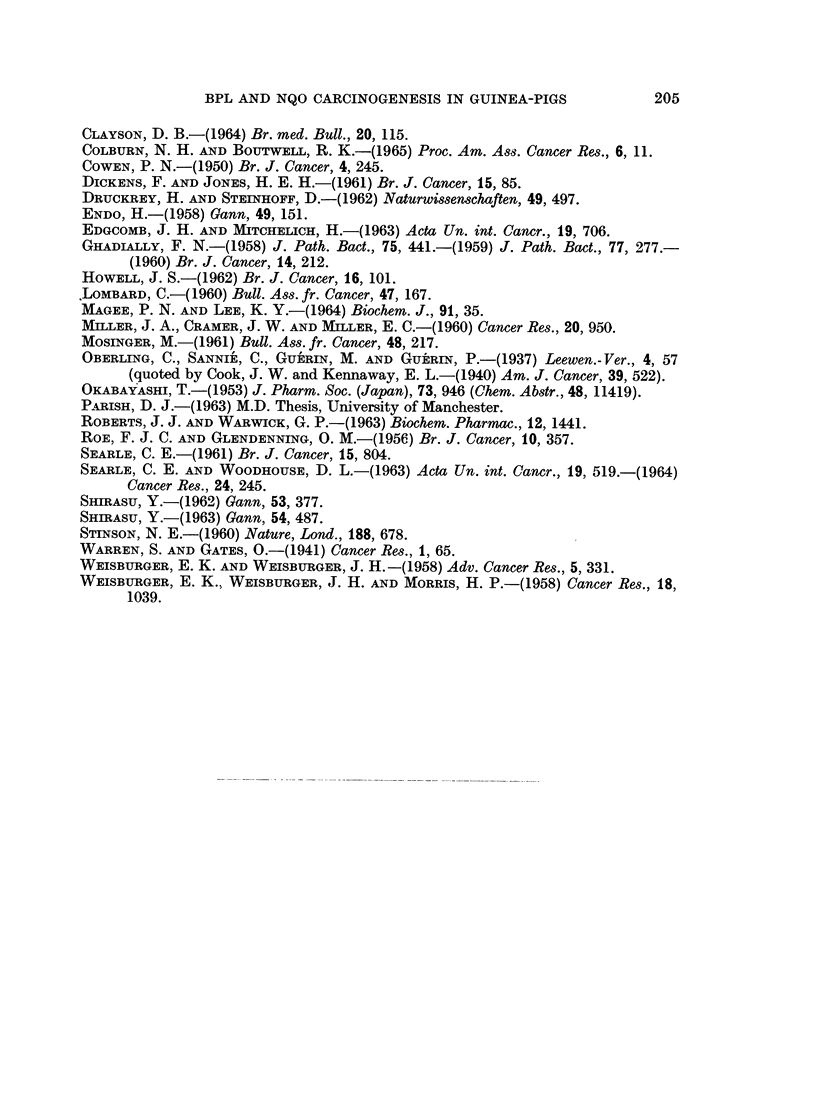# The carcinogenicity of beta-propiolactone and 4-nitroquinoline N-oxide for the skin of the guinea-pig.

**DOI:** 10.1038/bjc.1966.23

**Published:** 1966-03

**Authors:** D. J. Parish, C. E. Searle

## Abstract

**Images:**


### THE CARCINOGENICITY OF $\beta$-PROPIOLACTONE AND 4-NITROQUINOLINE N-OXIDE FOR THE SKIN OF THE GUINEA-PIG

D. J. PARISH* AND C. E. SEARLE

*From the Cancer Research Laboratories, Department of Pathology, The Medical School, Birmingham* 15

Received for publication November 25, 1965

A NUMBER of compounds which readily induce tumours in some other species of rodent fail to do so in the guinea-pig, and apart from some early papillomas observed after two years' treatment with benzo[*a*]pyrene (Oberling *et al.*, 1937) the only carcinogen so far reported to be active on skin application to guinea-pigs is 7,12-dimethylbenzo[*a*]anthracene (DMBA) (Berenblum, 1949; Edgcomb and Mitchelich, 1963).

We have now found, however, that a variety of tumours can be induced in the guinea-pig by skin applications of the carcinogens β-propiolactone (BPL) and 4-nitroquinoline N-oxide (NQO), provided treatment is continued for two or more years. The experiments are described in this paper, and the results are discussed in relation to " species resistance " and carcinogen metabolism.

### EXPERIMENTAL

### *Materials*

BPL (L. Light & Co.) was distilled under reduced pressure and applied in dry purified acetone as described by Searle (1961), and the NQO was used in acetone as described by Searle and Woodhouse (1964). Benzene was dried with potassium carbonate and redistilled.

### *Animals*

Guinea-pigs were obtained from the Department of Pharmacology, University of Birmingham ; their sex and colour were as shown in Table I. They were housed on sawdust in open runs, and were fed pellet diet SG. 1 and cabbage with free access to tap water.

The compounds were applied to four clipped areas, each about 3 sq. in. in area, on the front and rear flanks of each animal. Hair growth was slow on sites treated with NQO, but BPL-treated animals needed weekly clipping.

### *Treatment*

At first, BPL was applied twice weekly to two diagonally opposite sites in 2·5 per cent solution (v/v) and to the other two sites in 5·0 per cent solution. The standard volume was 0·5 ml. per site. Though Roe and Glendenning (1956) found that 5–10 per cent BPL in acetone caused ulceration and scarring of mouse skin, we found no such effect on our guinea-pigs, and after a few months the 5 per

* Present address: Department of Pathology, Royal Victoria Hospital, Bournemouth.

cent solution was used on all four sites. However, 5 per cent BPL in benzene proved too irritating, and the two animals treated with this solution for 5 weeks were, after a short break, transferred to the 5 per cent solution in acetone.

NQO was applied initially at 0·5 per cent in acetone to two diagonally opposite sites, and at 1·0 per cent to the other two sites, using 0·5 ml. per site twice weekly as with BPL. As was found when using lower concentrations of NQO in mice (as yet unpublished, Searle and Spencer), symptoms indicating a toxic effect gradually developed. A number of breaks in NQO treatment were therefore made to allow recovery, and after about 18 months of intermittent treatment, applications were continued at the two strengths on the right sites only.

Two animals on each compound died during the first year of treatment. The maximum period of survival from the start of treatment was 168 weeks with BPL and 134 weeks with NQO. Biopsies of skin lesions were carried out under ether anaesthesia on one BPL guinea-pig after 99 weeks, and on 3 NQO animals after 80, 103 and 119 weeks. Animals which died or were killed owing to poor condition were dissected, and treated skin, skin lesions and organs showing abnormal appearances were fixed (4 per cent formaldehyde-saline) and stained for histological examination with Ehrlich's haematoxylin-eosin and Weigert's iron haematoxylin-Van Gieson's stain. Suspected melanoma tissue was also stained by Masson's Fontana method. Details of these animals, their treatment and results are summarised in Table I.

TABLE I.—*Application of β-Propiolactone and 4-Nitroquinoline N-Oxide to Guinea-Pig Skin: Summary of Treatment and Results.*

| Compound | Animal and sex* | Biopsy (weeks) | Death (weeks) | Pathology |
|---|---|---|---|---|
| | 1 F. | — | 38 | — |
| | 10 M. | — | 46 | — |
| | 15 F.*† | — | 85 | Widespread pigmented naevi. |
| | 9 M.* | 99 | — | Keratoacanthoma; pigmented intradermal naevi. |
| $\beta$-Propiolactone in acetone | | — | 141 | Keratoacanthoma ; benign intradermal naevi ; hepatoma ; malignant tumour of lacrimal gland. |
| | 4 M. | — | 107 | Keratoacanthoma. |
| | 3 F.* | — | 119 | Very slight dermal naevi. |
| | 11 M. | — | 127 | — |
| | 14 M.† | — | 127 | Melanoma ; keratin cysts ; early naevi. |
| | 2 F. | — | 168 | — |
| | 13 F. | — | 2 | — |
| | 5 F. | — | 17 | — |
| | 12 F. | — | 56 | — |
| | 17 M.* | 80 | — | Sarcoma. |
| 4-Nitroquinoline N-oxide in acetone | | — | 126 | 4 Keratoacanthomas. |
| | 7 F. | 103 | — | 2 Trichoepitheliomas ; keratin cyst. |
| | | — | 123 | 3 Amelanotic melanomas ; metastases to lung and lymph nodes. |
| | 6 F. | — | 112 | — |
| | 16 M.† | 119 | — | Compound junctional naevus. |
| | | — | 128 | 2 Keratoacanthomas. |
| | 8 F. | — | 134 | Lymph node tumour ; keratin cyst. |

* The numbers are arbitrary numbers given to individual animals. Guinea-Pig No. 15 was brown and No. 17 black. All others were white with pink eyes except No. 3 and 9 which had dark eyes.

† Treated initially with benzene solutions of carcinogens.

### RESULTS

As will be seen by reference to Table I, the commonest tumour was the kerato-acanthoma. This tumour was induced by both BPL and NQO. Examples varied in complexity from simple squamous cysts to complex, flask-shaped tumours showing atypical squamous epithelial differentiation (Fig. 1–4). The range of appearances was similar to those described by other workers in a variety of species and using different carcinogenic agents (e.g. Ghadially, 1958, 1959, 1960 ; Howell, 1962).

Perhaps the most interesting tumours were those derived from pigment cells or melanocytes. These tumours are of particular pathological interest because the details of normal skin pigmentation in the guinea-pig (unlike many other commonly used experimental animals) closely resemble those of man, and furthermore the pigmented tumours produced in the guinea-pig resemble quite closely certain spontaneous tumours in man. In normal guinea-pig skin melanocytes are confined to the basal epidermal layers as in man. The first stage in tumour development appears to be a proliferation of these cells both in surface epidermis and in the stratified epithelium forming the hair follicles. Later, cells invade the dermis and in some examples activity in the basal epidermal layers may not be evident. These stages in development produce tumours which we have described as junctional, compound or intradermal pigmented naevi because of their similarity to the equivalent lesions in man (Fig. 5–7).

Malignant tumours of pigment cell origin—described as melanomas—have been separated from the previous tumours mainly on the basis of distinctive cytology, the component cells being much larger with an open nuclear pattern and frequently with a prominent eosinophilic nucleolus. Some examples show striking pleomorphism (Fig. 8 and 9). In these experiments only one such tumour was shown to be biologically malignant by the demonstration of metastases (Fig. 10–12) but in other experiments with DMBA a similar histological picture was also associated with metastases (Parish, 1963).

---

### EXPLANATION OF PLATES

FIG. 1.—Flask-shaped keratoacanthoma at top. More complex epidermal cyst at bottom. H. & E. ×10.

FIG. 2.—Superficial part of large epidermoid cyst lined by regular squamous epithelium. H & E. ×10.

FIG. 3.—Complex, multi-locular keratoacanthoma. H. & E. ×10.

FIG. 4.—Deep aspect of keratoacanthoma impinging on muscle layer. " Sebaceous " structures are frequently present in the cyst lining. H. & E. ×25.

FIG. 5.—Junctional naevus with groups of clear cells forming in the lower layers of the epidermis. At lower left is part of the wall of an epidermoid cyst. H. & E. ×100.

FIG. 6.—Detail of junctional activity. Packages of naevus cells are beginning to invade the dermis. H. & E. ×250.

FIG. 7.—Early compound naevus. Melanin is present in tumour cells and melanophages in the dermal component. Masson's Fontana. ×125.

FIG. 8.—Margin of malignant melanoma. A compound naevus is present in the upper right hand corner. H. & E. ×10.

FIG. 9.—Pleomorphic cytology of malignant melanoma with large nuclei containing prominent (eosinophilic) nucleoli. H. & E. ×250.

FIG. 10.—Metastasis from melanoma in lymph node. H. & E. ×100.

FIG. 11.—Metastases from melanoma in lung. H. & E. ×10.

FIG. 12.—Bizarre cytology of lung metastasis. H. & E. ×60.

1

2

3

4

Parish and Searle.

5

6

7

8

Parish and Searle.

9

10

11

12

Other isolated tumours which occurred in these animals included a hepatoma, a tumour of lacrimal gland, an undifferentiated skin tumour regarded as sarcoma, and a lymph node tumour which had histological appearances resembling secondary amelanotic melanoma, although we were unable to demonstrate a convincing primary skin tumour.

### DISCUSSION

In addition to its resistance to carcinogenesis by the polycyclic hydrocarbon carcinogens, referred to earlier in relation to skin carcinogenesis, the guinea-pig is resistant to various other agents which induce tumours in other rodents under suitable conditions. These include 2-amino- and 2-acetamido-fluorene (Weisburger and Weisburger, 1958), urethane (Cowen, 1950) and implanted plastic film (Stinson, 1960). There are also reports on the rarity of spontaneous tumours in the guinea-pig (Warren and Gates, 1941; Lombard, 1960 ; Mosinger, 1961).

However, a number of comparative studies on the metabolism of carcinogens suggest that the difference in response of the guinea-pig to some carcinogens compared with, e.g., the mouse may be attributable to differences in the way the substances are transported and metabolised rather than to an ill-defined " cancer resistance " in the guinea-pig.

In the case of the polycyclic hydrocarbon carcinogens, Chevallier, Manuel and Denoix (1946) noted that subcutaneously injected 3-methylcholanthrene and benzo[*a*]pyrene rapidly disappeared from the injection site in the guinea-pig, though they were detectable for many months in the rat. More recently, quantitative determinations of the amounts of carcinogenic hydrocarbons retained in the skin of different species two hours after application (Bock, 1963) have shown that guinea-pig skin retains much smaller amounts of the compounds than do mouse, rat and hamster skin. Bock's finding that DMBA was retained in considerably larger amounts than benzopyrene or methylcholanthrene accords with the fact that only DMBA has so far proved carcinogenic for guinea-pig skin.

Differences in the hydroxylation products of 2-acetamidofluorene formed in the guinea-pig and other species were demonstrated by Weisburger, Weisburger and Morris (1958), and the resistance of the guinea-pig to this carcinogen may be due to its failure to form the N-hydroxy metabolite (Miller, Cramer and Miller 1960). This type of compound, which is a substituted hydroxylamine, R.NHOH, may also be responsible for the activity of other aromatic amine carcinogens, a possibility discussed by Clayson (1964).

It is similarly possible that the hydroxylamine derivative of NQO, 4-hydroxyaminoquinoline N-oxide, formed in this case as one stage in the metabolic reduction of the nitro group, is of importance in NQO carcinogenesis, since it has been shown to be carcinogenic for mice on subcutaneous injection (Shirasu, 1963). That we have now found NQO to induce tumours in the guinea-pig does not conflict with the inactivity of acetamidofluorene because of the different modes of formation of the N-hydroxy metabolites in the two cases. Our results contrast, however, with those of Shirasu (1962) who obtained only one tumour after treating guinea-pigs with NQO by subcutaneous injection for up to 30 months.

The outstanding feature of the chemical behaviour of NQO is its extremely ready reaction in neutral solution with thiol-containing compounds, in which the labile nitro group is eliminated as nitrite (Okabayashi, 1953 ; Endo, 1958 ; Searle and Woodhouse, 1963). The failure of NQO to react under similar conditions with thiol-free compounds, in particular with nucleic acid derivatives, appears to favour the importance of metabolic reduction in NQO carcinogenesis, though the direct NQO-thiol reaction may well be responsible for the toxic effects observed by other workers and ourselves.

Unlike NQO, BPL reacts with a wide variety of organic groupings. It readily alkylates not only thiol groups, yielding for example S–2–carboxyethylcysteine by reaction with cysteine (Dickens and Jones, 1961), but also the 7-position of guanine derivatives (Roberts and Warwick, 1963), and there is now strong evidence linking the alkylation of guanine in mouse skin DNA by BPL with its carcinogenic action (Colburn and Boutwell, 1965). Other alkylating agents may thus also prove carcinogenic for the guinea-pig, provided that tests are continued long enough to allow for the longer life-span and generally slower rate of tumour development in this species. Diethylnitrosamine, which probably owes its carcinogenicity to alkylation of guanine by a metabolite (see, e.g., Magee and Lee, 1964), has in fact already been shown to be carcinogenic for the guinea-pig by Druckrey and Steinhoff (1962) and Argus and Hoch-Ligeti (1963).

The carcinogenicity of diethylnitrosamine, BPL and NQO for the guinea-pig, coupled with the results of the metabolic experiments referred to earlier, now form a strong body of evidence that the guinea-pig is not inherently resistant to chemical carcinogenesis. Its apparent resistance to some agents is due to its relatively slow rate of tumour development, to rapid elimination of some carcinogens or to failure to form carcinogenic metabolites.

The guinea-pig differs nevertheless from other generally used experimental animals in details of normal skin pigmentation and of pigmented tumours, which bear a close resemblance to those seen in man. This feature, described in the Results section, makes the guinea-pig a potentially useful model for the study of melanotic tumours of the type which occur in man.

### SUMMARY

1. The carcinogens $\beta$-propiolactone and 4-nitroquinoline N-oxide have been applied for long periods to the skin of guinea-pigs.

2. Some guinea-pigs treated with each compound eventually developed pigmented or non-pigmented lesions of the skin. One animal treated with the lactone had an extensive hepatoma.

3. The results are discussed with regard to carcinogen metabolism and the resistance of the guinea-pig to some carcinogens.

4. The similarity in the pigmentation of normal skin and pigmented tumours between the guinea-pig and man is emphasised.

The work was supported by the Birmingham Branch of the British Empire Cancer Campaign for Research.

### REFERENCES

ARGUS, M. F. AND HOCH-LIGETI, C.—(1963) *J. natn. Cancer Inst.*, **30**, 533.

BERENBLUM, I.—(1949) *J. natn. Cancer Inst.*, **10**, 167.

BOCK, F. G.—(1963) *Natn. Cancer Inst. Monogr.*, No. 10, 361.

CHEVALLIER, A., MANUEL, S. AND DENOIX, P.—(1946) *Bull. Ass. fr. Étude Cancer*, **33**, 223.

CLAYSON, D. B.—(1964) *Br. med. Bull.*, **20**, 115.
COLBURN, N. H. AND BOUTWELL, R. K.—(1965) *Proc. Am. Ass. Cancer Res.*, **6**, 11.
COWEN, P. N.—(1950) *Br. J. Cancer*, **4**, 245.
DICKENS, F. AND JONES, H. E. H.—(1961) *Br. J. Cancer*, **15**, 85.
DRUCKREY, H. AND STEINHOFF, D.—(1962) *Naturwissenschaften*, **49**, 497.
ENDO, H.—(1958) *Gann*, **49**, 151.
EDGCOMB, J. H. AND MITCHELICH, H.—(1963) *Acta Un. int. Cancr.*, **19**, 706.
GHADIALLY, F. N.—(1958) *J. Path. Bact.*, **75**, 441.—(1959) *J. Path. Bact.*, **77**, 277.—(1960) *Br. J. Cancer*, **14**, 212.
HOWELL, J. S.—(1962) *Br. J. Cancer*, **16**, 101.
LOMBARD, C.—(1960) *Bull. Ass. fr. Cancer*, **47**, 167.
MAGEE, P. N. AND LEE, K. Y.—(1964) *Biochem. J.*, **91**, 35.
MILLER, J. A., CRAMER, J. W. AND MILLER, E. C.—(1960) *Cancer Res.*, **20**, 950.
MOSINGER, M.—(1961) *Bull. Ass. fr. Cancer*, **48**, 217.
OBERLING, C., SANNIÉ, C., GUÉRIN, M. AND GUÉRIN, P.—(1937) *Leewen.-Ver.*, **4**, 57 (quoted by Cook, J. W. and Kennaway, E. L.—(1940) *Am. J. Cancer*, **39**, 522).
OKABAYASHI, T.—(1953) *J. Pharm. Soc.* (*Japan*), **73**, 946 (*Chem. Abstr.*, **48**, 11419).
PARISH, D. J.—(1963) M.D. Thesis, University of Manchester.
ROBERTS, J. J. AND WARWICK, G. P.—(1963) *Biochem. Pharmac.*, **12**, 1441.
ROE, F. J. C. AND GLENDENNING, O. M.—(1956) *Br. J. Cancer*, **10**, 357.
SEARLE, C. E.—(1961) *Br. J. Cancer*, **15**, 804.
SEARLE, C. E. AND WOODHOUSE, D. L.—(1963) *Acta Un. int. Cancr.*, **19**, 519.—(1964) *Cancer Res.*, **24**, 245.
SHIRASU, Y.—(1962) *Gann*, **53**, 377.
SHIRASU, Y.—(1963) *Gann*, **54**, 487.
STINSON, N. E.—(1960) *Nature, Lond.*, **188**, 678.
WARREN, S. AND GATES, O.—(1941) *Cancer Res.*, **1**, 65.
WEISBURGER, E. K. AND WEISBURGER, J. H.—(1958) *Adv. Cancer Res.*, **5**, 331.
WEISBURGER, E. K., WEISBURGER, J. H. AND MORRIS, H. P.—(1958) *Cancer Res.*, **18**, 1039.